# Diversity, Community Composition and Abundance of Anammox Bacteria in Sediments of the North Marginal Seas of China

**DOI:** 10.1264/jsme2.ME15140

**Published:** 2016-05-14

**Authors:** Ahmed Shehzad, Jiwen Liu, Min Yu, Shakeela Qismat, Jingli Liu, Xiao-Hua Zhang

**Affiliations:** 1 College of Marine Life Sciences, Ocean University of ChinaQingdao 266003China; 2 Institute of Evolution and Marine Biodiversity, Ocean University of ChinaQingdao 266003China

**Keywords:** anammox bacteria, novel phylotypes, marginal seas, physicochemical responses

## Abstract

Over the past few decades, anammox bacteria have been recognized as key players that contribute significantly to the release of large amounts of nitrogen in the global marine nitrogen cycle. In the present study, the diversity, community composition, and abundance of anammox bacteria from the sediments of four diverse regions in the north marginal seas in China were determined via clone library construction and a quantitative PCR analysis. The clone libraries retrieved by the 16S rRNA gene and *Hzo* gene markers indicated that “*Candidatus* Scalindua” was the predominant group throughout the sites examined. The 16S rRNA gene clone libraries revealed exceptional diversity by identifying two potential novel anammox clades, as evidenced by the high sequence similarities between these two clades and known anammox genera, and their unique phylogenetic positions with high bootstrap values. However, their potential roles in the anammox reaction need to be validated. Six novel members of *Planctomycetes*, divergent from the known genera of anammox bacteria, were also detected. A phylogenetic analysis by Hzo protein sequences revealed the existence of two known genera, *i.e.*, “*Candidatus* Jettenia” and “*Candidatus* Anammoxoglobus”, which are rarely captured from marine sediments. Among all ecological parameters investigated, the distribution patterns and composition of anammox bacteria were found to be influenced by salinity, total organic matter, and temperature. The abundance of the anammox bacterial 16S rRNA gene from the sites examined ranged between 3.95×10^5^ and 9.21×10^5^ copies g^−1^ wet sediment and positively correlated with the median size of the sediment sample.

The load of nitrogen around the globe has been amplified enormously due to unchecked anthropogenic activities (13). In the marine ecosystem, anammox processes have been shown to play a significant role in anaerobic N_2_ production (43), and may be responsible for the removal of between 30 and 70% of fixed N (10). The importance of anammox bacteria in the global nitrogen cycle has attracted researchers interested in their community and functional dynamics (11, 32, 48, 50).

Anammox bacteria are the inhabitants of diverse natural environments, including hypoxic or anoxic water columns, marine sediments, estuarine sediments, anoxic tropical freshwater, continental margin sediments, activated sludge, multi-year sea ice, and geothermal subterranean oil reservoirs (11, 16, 24, 32, 36, 39, 48, 50). Strong anammox activity was first described in the sediments of the Baltic-North Sea, in which anammox accounts for up to 67% of anaerobic N_2_ production (47). Subsequent studies established through extensive research that anammox bacteria have the ability to survive in a wide range of environments with diverse temperature adaptability ranging from psychrophilic to mesophilic, thermophilic, and even hyperthermophilic environments (4, 12).

Anammox bacteria are chemolithoautotrophs, and all of them are affiliated to the order “*Candidatus* Brocadiales” within the phylum *Planctomycetes* (21). Five genera of anammox bacteria have been identified: “*Ca.* Brocadia”, “*Ca.* Kuenenia”, “*Ca.* Anammoxoglobus”, “*Ca.* Jettenia”, and “*Ca.* Scalindua” (42). Moreover, almost nine species of *Scalindua* lineages have been documented in previous studies with different origins (3, 6, 36). In marine environments, *Scalindua* was found to be the dominant group identified with enormous diversity by employing 16S rRNA and hydrazine oxidoreductase (Hzo) gene biomarkers (3). Although several non-*Scalindua* anammox bacteria have also been identified (1, 4–6, 15), it is not confirmed whether these non-*Scalindua* anammox bacteria are core components of marine microorganisms or restricted to particular marine environments.

Anammox bacteria hitherto are not isolated in pure cultures due to their complex nutrient and physical requirements. Therefore, molecular methods such as 16S rRNA gene amplification and DNA probing techniques (38) are considered valuable in the study of environmental anammox bacteria; however, most of the primers used in studies are less targeted and difficult to design (15). All anammox bacteria can encode hydrazine synthase, which catalyzes the production of hydrazine from NH_4_^+^ and NO (14). Similarly, *hzo*, also known as hydrazine dehydrogenase (*hdh*), is a significant part of the anammox bacterial genome, which oxidizes hydrazine to N_2_ (20). The utilization of 16S rRNA and functional genes as key biomarkers of anammox bacteria has become a routine technique in comprehensive ecological studies (7, 15, 25), which have provided insights into the ecological role of anammox bacteria in nitrogen cycles (6, 24).

The north marginal seas of China receive high loads of nutrients from the land, particularly from the Yangtze and Yellow Rivers, and consequently, appear to significantly contribute to the biogeochemical cycles of the coastal ecosystem. Bohai Sea (BS) is the innermost bay of China, with an area of 7.7×10^4^ km^2^ and an average depth of 18 m. It is linked to the North Yellow Sea (NYS) through the narrow Bohai channel and receives massive expulsions of terrestrial materials including nutrients and sediment from more than 40 rivers, mainly the Yellow River (6). The Yellow Sea (YS), which is a semi-enclosed sea and part of the China inland, is located along the Korean Peninsula. The total area of YS is approximately 3.8×10^5^ km^2^, and is divided into the South Yellow Sea (SYS) and NYS by a boundary starting from Chengshan Cape, China and along Changshanchuan Island, Korea (28). The East China Sea (ECS) from the west is linked to mainland China and to the east by a chain of islands in the western Pacific Ocean (51). On the west, saline and the warm boundary of Kuroshio closely intermingles with the eastern outer ridge of ECS, while there is a huge entrance of freshwater from the Yangtze River to ECS (2). The discharge of the Yangtze River may also have a marked effect on the sedimentation of SYS (23).

The immense inputs from the Yangtze and Yellow Rivers to the north marginal seas in China were previously suggested to have caused significant differences in physicochemical features at different sea areas (27, 29). Thus, a comprehensive comparison of anammox bacterial assemblages among different areas is needed in order to shed light on their ecological roles in the north marginal seas in China, despite that some data related to anammox bacterial communities are available in individual areas of ECS or BS (6, 41). Therefore, the aim of the present study was to study the diversity, composition, and abundance of anammox bacteria from 12 sampling sites in four north marginal seas located along the coastal line of China. This study focused on determining whether it was possible to expose some discrete anammox bacterial communities, and investigated the role of the resident anammox bacterial community in the nitrogen cycle together with the impact of ecological parameters on their distribution patterns and abundance in the studied environment. The results obtained may be beneficial for obtaining a deeper understanding of the comparative role of the anammox bacterial community in such a vast marine environment.

## Materials and Methods

### Sampling and physiochemical analysis

**Fig. 1:**
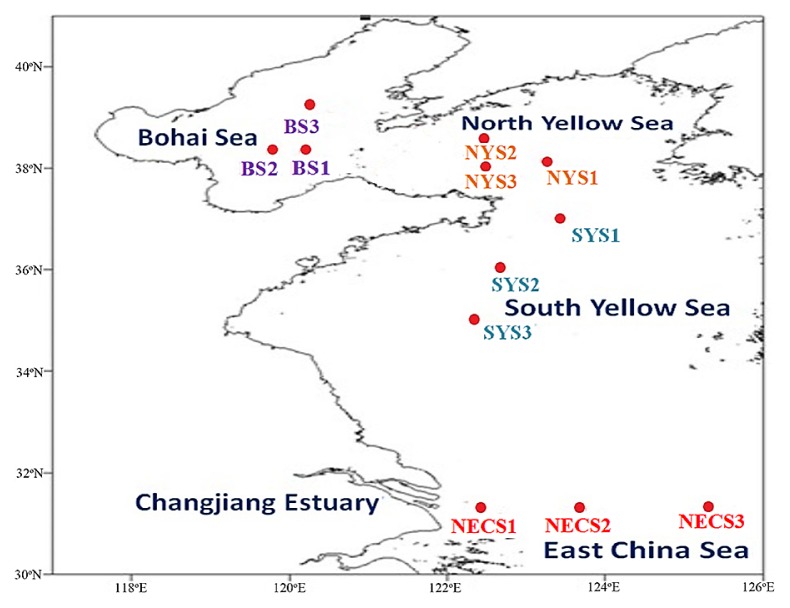
Map indicating sampling locations across four marginal seas in China.

Twelve sediment samples were collected from four north marginal seas in China with three sampling sites in each marginal sea. These selected areas included BS (BS1, BS2, and BS3), NYS (NYS1, NYS2, and NYS3), SYS (SYS1, SYS2, and SYS3), and NECS (NECS1, NECS2, and NECS3). The collection of sediment samples was performed during a cruise by *R/V* ‘*Dong Fang Hong 2*’ between 29 Apr and 22 May, 2012 (Fig. 1, [27]), using a box corer onboard. A 5-mL syringe (luer end removable) was used to collect surface sediments (0–2 cm). Samples used for DNA extraction were frozen in liquid nitrogen and those for median size and total organic matter (TOM) determinations were persevered at −20°C; the sediment samples used for the detection of chlorophyll *a* were wrapped in aluminum foil before being stored at −20°C. Physicochemical parameters such as salinity, temperature, pH, dissolved oxygen (DO), and depth of the bottom water (typically 1–2 m above the sediments) were estimated by Seabird 911 Conductivity-Temperature-Depth (CTD). Measurements of sediment water content, TOM, and Chl *a* were performed using methods described previously by Liu *et al.* (30).

### DNA extraction, amplification, cloning, sequencing, and phylogenetic analysis

Initially, 0.3 g of sediment was used to extract total genomic DNA by the Power Soil DNA Kit (Mol Bio Laboratories, Carlsbad, CA, USA), according to the manufacturer’s protocol with slight modifications. The concentration of extracted DNA from sediment samples was measured by a NanoDrop 2000 (Thermo Scientific, Wilmington, DE, USA) spectrophotometer. Anammox 16S rRNA and *hzo* genes were used to amplify the desired gene fragments by a nested PCR technique described previously (5, 15) using the primers PLA46f-1390r-AMX368f-820r and *hzo*AB1F/R-*hzo*AB4F/R, respectively.

The obtained product was separated by electrophoresis on a 1% agarose gel and purified using the Agarose Gel DNA Recovery Kit (Biomed, Beijing, China) according to the manual of the manufacturer. The purified amplicons were ligated into the pUCm-T vector (Sangon Biotech, Shanghai, China) and transformed into the competent *Escherichia coli* JM109 strain prepared in the lab. The transformed *E. coli* cells with plasmid insert-positive recombinants were carefully selected using X-Gal-IPTG LB indicator plates supplemented with 100 μg mL^−1^ ampicillin, and the expected transformants were re-amplified by the colony PCR technique using M-13F/R primers. In order to confirm the correct size of the cloned fragment, the colony PCR product was run on a 1% agarose gel by electrophoresis in order to avoid erroneous fragment assortment for sequencing.

The retrieved 16S rRNA gene sequences and amino acid sequences translated from *hzo* gene sequences were aligned by Clustal-X (version 2.1) (49). 16S rRNA gene sequences and amino acid sequences with 97% identity were both grouped into operational taxonomic units (OTUs) using the Dotur program (35). The phylogenetic tree was constructed by the neighbor-joining algorithm (34) with Kimura-2 parameters and P-distance methods, respectively (22), followed by 1,000 bootstrap replicates using the MEGA software (version 5.2) (46).

In order to confirm the identity of anammox bacteria, all the sequences obtained were blasted against the National Center for Biotechnology Information (NCBI) database. The 16S rRNA and *hzo* gene sequences of anammox bacteria obtained in this study are available in the NCBI database under the accession numbers KM266322 to KM266372 and KP273918 to KP273969, respectively.

### Quantitative PCR assay

The abundance of anammox bacteria was investigated using the qPCR system as described previously (5, 12). The quantification of anammox bacteria in sediment samples was studied by qPCR in triplicate. AMX-808-F and AMX-1040-R primers (5) were used with an ABI 7500 sequence detection system (Applied Biosystems, Foster City, CA, USA) employing the SYBR green method (8, 9). The competent JM109 strain of *E. coli* was prepared in the lab and used for the cloning of anammox 16S rRNA genes inserted into the pUCm-T vectors. The purified plasmid DNA products were quantified using Quant-iT PicoGreen double-stranded DNA assays (Invitrogen, Carlsbad, CA, USA). The 20-μL PCR mixture contained 10 μL of 2×SYBR Premix II (TaKaRa, Otsu, Japan), 6 μL of H_2_O, 0.8 μL of each primer (10 mM), 0.4 μL of ROX reference dye II, and 2 μL of template DNA. All reactions were performed in eight-strip thin-well PCR tubes with ultraclean cap strips (ABgene, United Kingdom). The qPCR protocol was performed as follows: 95°C for 3 min, followed by 45 cycles at 95°C for 30 s, 55°C for 30 s, and 72°C for 30 s. Standard curves were obtained with ten-fold serial dilutions of standard plasmids, and melting curves and gel electrophoresis were performed in order to confirm the specificity of the qPCR amplification. Cycle thresholds were determined by comparisons with the standard curves. In all experiments, negative controls containing no template DNA were performed under the same qPCR procedure in order to avoid possible contamination. The qPCR product of anammox bacteria was validated by sequencing, and is available in the NCBI database under the accession number KU315118.

### Statistical analysis

By using Dotur, Chao 1, Shannon indices, a rarefaction analysis, and the numbers of observed OTUs were calculated for each gene library in order to determine the diversity of anammox bacteria. The topographical distribution of the phylogenetic structure of anammox bacterial assemblages and their relationships with ecological parameters were analyzed through the online software UniFrac by a Jackknife analysis, principal coordinate analysis (PCoA) (31), and canonical correspondence analysis (CCA) employing CANOCO software (version 4.5). The relationship between environmental factors and the abundance of anammox bacteria in the study sites was estimated by R Package (19). The sequences associated with novel anammox genera and *Planctomycetes* were excluded in all analyses due to ambiguities in their classification, except for the phylogenetic analysis.

## Results

### Characterization of sediment in study sites

The different physicochemical parameters of the collected samples have been measured and described (27). Briefly, the temperature of water samples from NECS was significantly higher than those from NYS (*P*=0.024). Similarly, salinity was lower in sediments from BS, NYS, and NECS1 (26.88 psu) than in those from the other sites (*P*=0.044). DO in bottom water and the median size of the sediment sample were higher in BS and NYS than those in SYS and NECS. The amount of Chl *a*, ranging between 0.08 and 0.81 μg g^−1^, was less in the sediment from NECS than in those from the other three north marginal seas in China (27).

### Amplification of anammox bacteria

Twelve gene libraries were constructed from the surface sediments of distinct sampling sites in the north marginal seas of China. A total of 529 sequences with 16S rRNA gene primers and 505 sequences by *hzo* gene primers were used in the present study. The final products amplified had average sizes of 477 bp and 600 bp for 16S rRNA and *hzo* gene sequences, respectively.

### Anammox phylogeny by 16S rRNA gene sequences

**Fig. 2:**
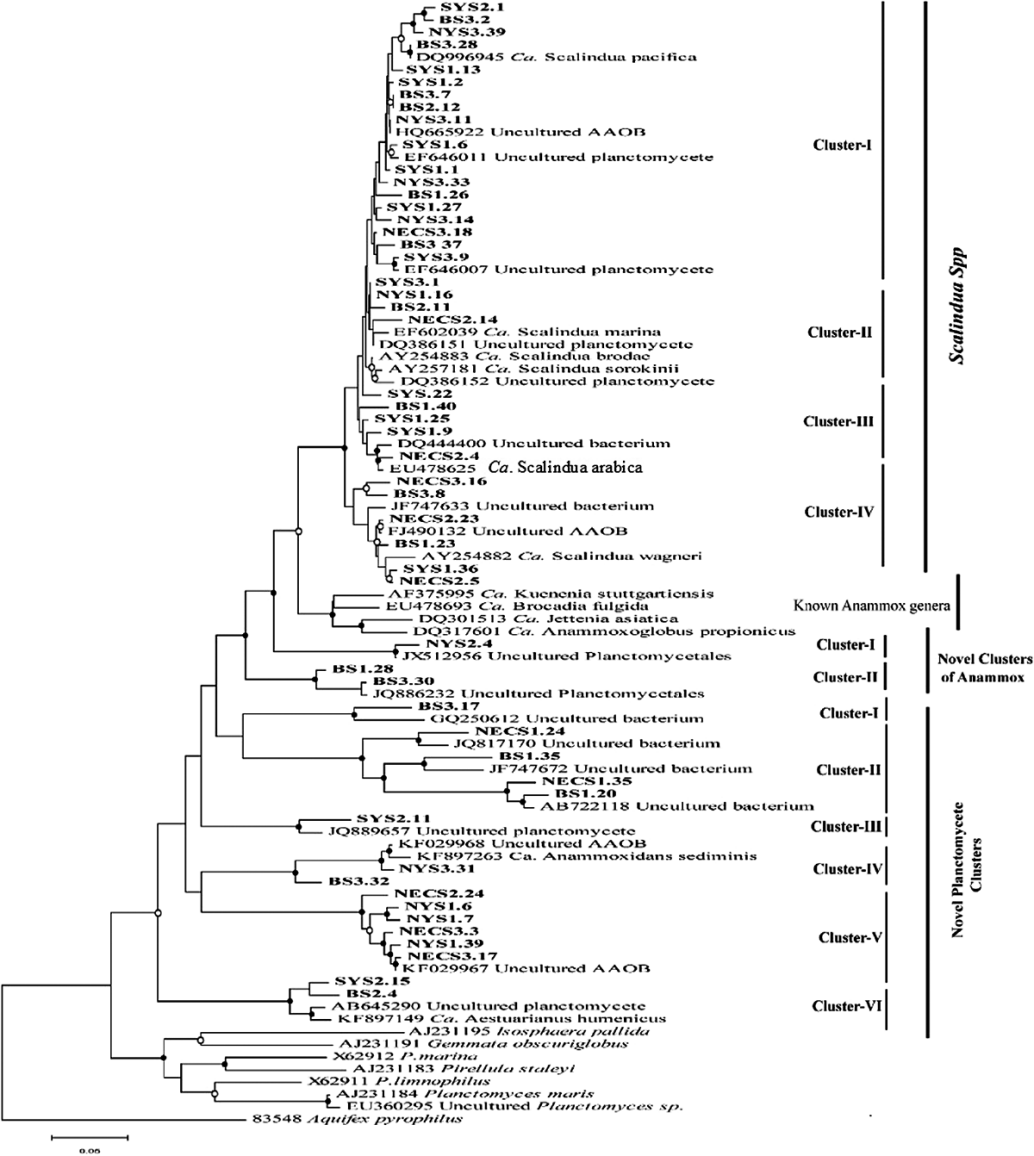
Phylogenetic tree of anammox bacterial 16S rRNA genes recovered from north marginal seas in China. The 16S rRNA gene sequences retrieved during the investigation and through public databases were aligned with the Clustal X program and a phylogenetic tree was constructed after a neighbor-joining analysis (MEGA-version 5.2). Bootstrap values (*n*=1,000 replicates) representing the solid circle symbols with greater than 70%, whereas equal to or greater than 50% or less than 70% are shown with open circle symbols on the relevant nodes. The substitution rate of nucleotides is characterized by the distance of the tree branch, and the expected number of changes per homologous position is signified by the scale bar. *Aquifex pyrophilus* (M83548) was used as the out-group.

A phylogenetic analysis of the amplified 16S rRNA gene sequences was performed in order to classify different members of anammox bacteria. The 52 unique phylotypes defined at a 3% cut-off value of nucleotide variation were selected for the better representation of 16S rRNA sequences. The retrieved results showed that *Scalindua* was the most dominant group, comprising 86.20% of all sequences probed. However, the remaining sequences (approximately 13.80%) were distantly related to any known genera of anammox bacteria. The phylogenetic analysis showed 12 distinct clusters including four clusters (Fig. 2) affiliated to the previously reported *Scalindua* species, and eight novel clusters with discrete positions in the consensus tree.

The retrieved 16S rRNA gene sequences in *Scalindua* Clusters I and II were associated with “*Ca.* Scalindua pacifica”, “*Ca.* Scalindua mirina”, “*Ca.* Scalindua sorokonii”, and “*Ca.* Scalindua brodae”, with similarity levels of 94.6–99.4%, 93.4–98.6%, 94.8–98.6%, and 96.3%–99.1%, respectively, and their closest sequences were recovered in sediments from the South China Sea, Gullmar fjord, Sweden, Black Sea, and wastewater treatment plant landfill leachate in Pitsea (40). The sequences of this study belonging to *Scalindua* Clusters III and IV were affiliated with “*Ca.* Scalindua arabica” and “*Ca.* Scalindua wagneri”, and showed similarity levels of 90.4–97.7% and 90–97.7%, respectively, with the sequences recovered from the oxygen minimum zone of the Arabian Sea and wastewater treatment plant landfill leachate in Pitsea (36).

The eight novel clades were discovered from all four marginal seas, predominantly from the sediment of NECS. They comprised 19 phylotypes and did not cluster with any of the identified anammox genera. However, the Blast results against the NCBI database along with distance-based similarity level results showed close relationships between some of the novel clades with the anammox lineage. In this context, two of the novel clusters may represent potential new genera of anammox bacterial assemblages (Fig. 2).

Novel Anammox Cluster I included a single phylotype, which displayed a close relationship (98.5% similarity) to the sequence recovered from the arctic sediment (Tait, unpublished data; direct GenBank submission). It showed a less than 89% affiliation with all of the well-defined anammox bacterial sequences in the tree. Novel Anammox Cluster II comprised two phylotypes with sequence similarities of 94% and 98% to the sequences retrieved from freshwater wetland sediments (Wang and Gu, unpublished; direct GenBank submission), respectively. The sequence similarities of the two phylotypes with the remaining identified anammox sequences were found to be 82.7%–88.4 and 83.3–88.4%, with <95% sequence similarity to each other. The high bootstrap values (>70%) in the phylogenetic tree between the clusters of these two novel clades and known anammox genera further indicates that they represent potential novel anammox members. However, the remaining six novel clusters, due to their ambiguous placing in the tree as well as the Blast results and distance-based similarity level, were found to be novel members of *Planctomycetes* instead of novel groups of anammox bacteria. Novel *Planctomycetes* Clusters I and II consisted of five phylotypes with affiliations to the sequences obtained from a sea water column in the Gulf of Mexico, sub-seafloor massive sulfide deposit, sub-seafloor sediment at the Good Weather Ridge, and Manantial del Toro hypersaline Groundwater (Ye *et al.*; Kato *et al.*; Lai., and Macalady *et al.*, unpublished data; direct GenBank submission) with 91.1%, 62–96.7%, 69.6–93.1%, and 68–84% sequence similarities, respectively. *Planctomycetes* Clusters III (one phylotype) and IV (two phylotypes) had 95% and 95–99% similarities with the sequence obtained from the sediments of the Yangtze River Estuary and Zhoushan Island (Hou and Zheng, and Zhang *et al.*, unpublished data; direct GenBank submission), respectively. *Planctomycetes* Clusters V and VI with six and two phylotypes, respectively, showed similarity levels of 91.6–96.0%, 92.5–99.7%, and 94.7–98.8% to the sequences recovered from the marine sediments of Zhoushan Island and Shimokita Peninsula, and sediments from Pearl Estuary (Zhang *et al.*, and Nunoura *et al.*, unpublished data; direct GenBank submission) (12), respectively.

### Anammox phylogeny by Hzo protein sequences

**Fig. 3:**
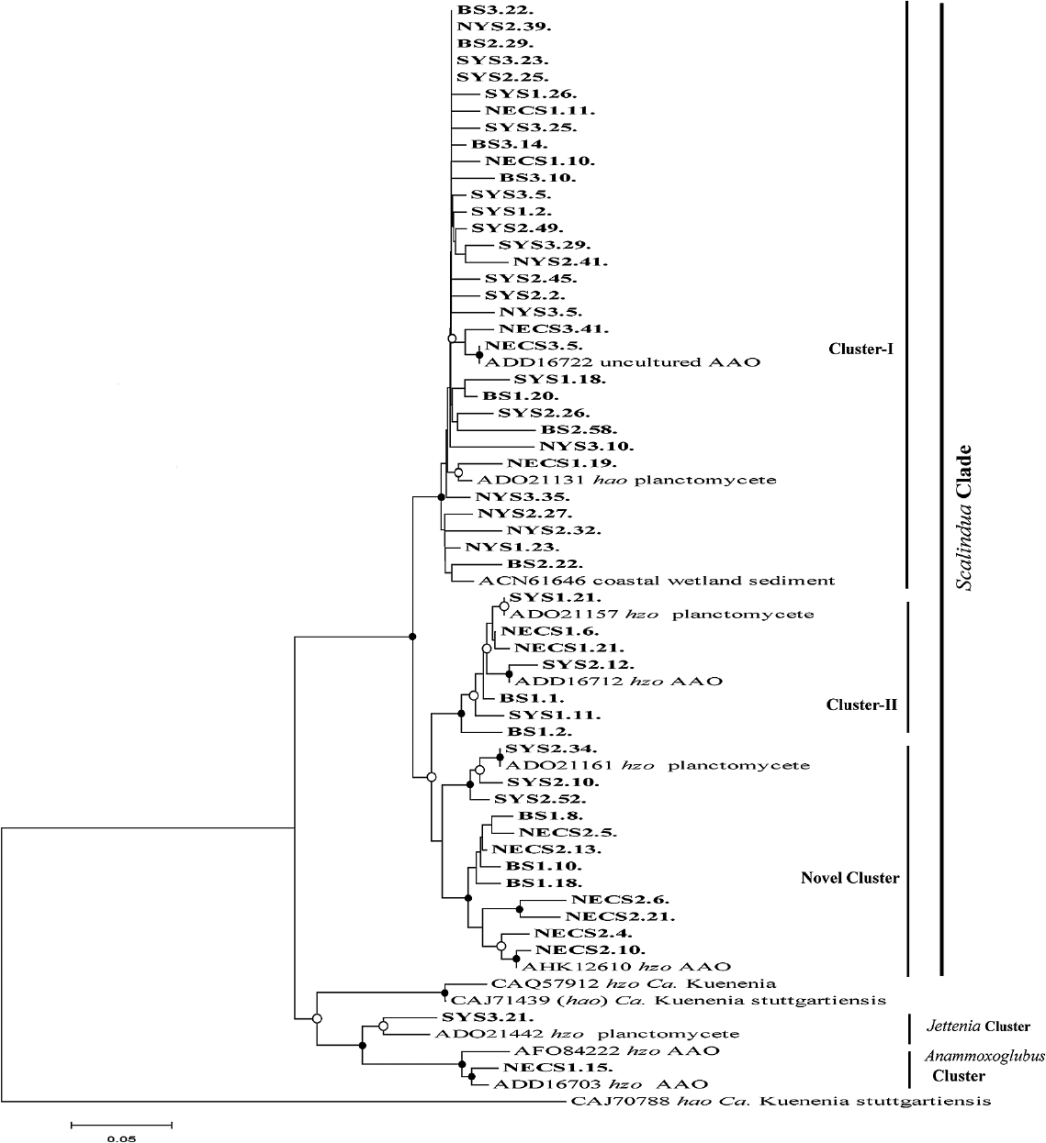
Phylogenetic tree of anammox bacterial Hzo protein sequences deduced from recovered *hzo* gene sequences from north marginal seas in China. The deduced Hzo protein sequences recovered during the investigation and through public databases were aligned with the Clustal X program and a phylogenetic tree was constructed after a neighbor-joining analysis (MEGA-version 5.2). Bootstrap values (*n*=1,000 replicates) represent the solid circle symbols with greater than 70%, whereas equal to or greater than 50% or less than 70% are shown with open circle symbols on the corresponding nodes. The substitution rate of nucleotides is characterized by the distance of the tree branch, and the expected number of changes per homologous position is signified by the scale bar. *Candidatus* Kuenenia stuttgartiensis (CAJ70788) was used as the out-group.

The phylogenetic tree constructed from the deduced Hzo protein sequences also indicated the high diversity of the anammox bacterial community in sediments from the north marginal seas in China. Fifty-three phylotypes were defined at a 3% cut-off value of protein variation. A total of 99.16% of sequences belonged to the *Scalindua* group, whereas only 0.84% was related to the two other genera of anammox bacteria. The whole phylogenetic tree was classified into three *Scalindua*-related clusters (including a novel cluster) and two clusters affiliated with *Ca.* Jettenia and *Ca.* Anammoxoglobus (Fig. 3). The predominant and widely distributed cluster I was phylogenetically associated with the *Scalindua* group and covered 87.5% of all sequences of the gene libraries used in the consensus tree.

The Hzo protein sequences assembled in Clusters I and II shared 95–100%, 94–98%, 92–97%, 96.6–98.60%, and 95.1–100% identities with those recovered from hypernutrified Jiaozhou Bay, sediments from deep sea hydrothermal vents, deep sea tephra deposits, and surface sediments from the coastal wetland at the Mai Po Nature Reserve (7, 25). However, the novel clade (consisting of 12 phylotypes) positioned in the *Scalindua* group showed less than 98% similarity to the other sequences, *i.e.*, 74.5–95.6%, and may represent a new clade of the *Scalindua* genus. The phylotypes of novel clades showed close similarities to the sequences recovered from deep sea tephra deposits and subsurface sediments from Pearl Estuary with identity levels of 95–98.7% and 91.7–95% (12, 15). The *Anammoxoglobus* and *Jettenia* clades represented the smallest fractions of the total Hzo protein sequences (approximately 0.84%) and showed a close affiliation with the protein sequences recovered from the sediments of Dongjiang River and Black River (45). The phylotypes representing the *Jettenia* and *Anammoxoglobus* clades shared sequence identities of 95.1% and 95.1–97.1% to their closest counterparts, respectively. The overall phylotypes obtained from protein sequences were 82.1–98.8% identical to each other and shared 92–100% similarities to the closest sequences in GenBank.

### Anammox bacterial diversity and distribution based on 16S rRNA gene and *hzo* gene sequences

The overall diversity of anammox bacteria with 16S rRNA gene sequences was higher than that calculated from Hzo protein sequences (Table 1). The highest diversity of anammox bacteria was estimated from the BS1 site, whereas site SYS3 had the lowest diversity with 16S rRNA gene sequences (Table 1, Fig. S1). Based on the deduced Hzo amino acid sequences, the highest diversity was detected in sediment from NECS2, while the lowest was in sediment from SYS3. “*Ca.* Scalindua pacifica” (*Scalindua*-Cluster I) was ubiquitous throughout the study sites, signifying 23% of all sequences obtained in the study. Within *Scalindua* Cluster II, “*Ca.* Scalindua marina” was found to be diverse throughout the areas studied, accounting for 33% of all gene sequences, particularly in sediment from NYS (57%); similarly, “*Ca.* Scalindua brodae/sorokinii”, also belonging to *Scalindua* Cluster II, each accounted for 15% of all gene sequences. However, the rarely identified “*Ca.* Scalindua wagneri” (*Scalindua*-Cluster IV) and “*Ca.* Scalindua arabica” (*Scalindua*-Cluster III) were estimated to account for 5.5% and 8.5%, respectively. The relative abundance of the *Scalindua* clusters was not consistent across the sites studied from the same sampling area (Fig. S2a), which indicated that the distribution of different *Scalindua* species was not unique in each separated sea area in the north marginal seas of China. More than 50% of the sequences related to novel clades were recovered from NECS, whereas almost 30% were detected in sediment from NYS. The high prevalence and dominance of the *Scalindua* lineage was also determined with *hzo* gene sequences from all sediment samples (Fig. S2b).

**Fig. 4:**
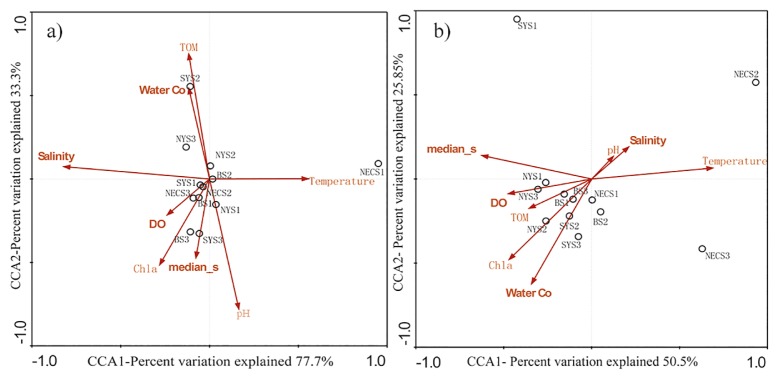
CCA ordination plots for the first two dimensions to display the relationship between anammox bacterial diversity and environmental factors using a) 16S rRNA gene sequences and b) Hzo protein sequences retrieved from sediments in four areas in north marginal seas in China. The relationship between environmental parameters and CCA axes are represented by the lengths and angles of the arrows.

The anammox bacterial distribution determined by 16S rRNA and deduced Hzo amino acid sequences and their relationship with ecological parameters were examined using CCA. TOM (*P*=0.008) and salinity (*P*=0.044) significantly contributed to the spatial distribution of anammox bacteria (1,000 Monte Carlo permutations) with 16S rRNA sequences. CCA1 clearly distinguished anammox bacterial assemblages in sediment from NECS1 from the other sites, explaining 77.7% of the cumulative variance of the anammox bacterium-environment relationship (Fig. 4a). The rest of the sites exhibited homogeneity, and the ecological parameters used in the present study did not have a significant impact on the spatial distribution of anammox bacteria in the environment.

Data analyzed with Hzo protein sequences revealed that temperature had a marked impact and was proven to be the only influential environmental parameter that correlated with variations in the anammox bacterial community structure (*P*=0.024). CCA1, which explained 50.5% of the cumulative variance of the anammox bacterial relationship with the environment, separated the anammox bacterial assemblages in sediment from NECS2 from that in sediment from the other sites. SYS1 and NECS3 also had dissimilar distribution patterns of the anammox bacterial community along with CCA2, which explained 25.85% of the cumulative variance of the anammox bacterial relationship with the environment (Fig. 4b). The community composition and distribution of anammox bacterial assemblages in the rest of the sampling sites showed homogeneity.

UniFrac environmental clustering by the Jackknife analysis with 16S rRNA gene sequences showed that the anammox assemblages of each sampling area were not clearly distinct (Fig. S3a). In contrast, the anammox assemblages of NECS2, NECS3, BS1, and SYS1 had heterogeneity in the distribution of anammox assemblages with the rest of the sites in the study when determined by the PCoA analysis (Fig. S4a). Similarly, jackknife clustering and PCoA both showed that the *hzo* gene sequences of the anammox assemblages in sediment from NECS1 and NECS2 were distant from the rest of the stations (Fig. S3b and S4b). These results demonstrated that the anammox assemblages had no significant dissimilarities among most of the samples examined and they displayed a homogeneous distribution in each sea area of the Chinese marginal seas.

### Abundance of anammox bacteria

The amplification efficiency of anammox bacterial 16S rRNA genes was estimated to be 102%, and the standard curves displayed a clear linear relationship (R^2^=0.996) indicating high primer hybridization and extension efficiency (Table S1). According to the amplification results, the highest abundance was determined from SYS2 with up to 9.21×10^5^ copies g^−1^ sediment. SYS1 had the lowest abundance at 3.95×10^5^ copies g^−1^ sediment (Table S3). Pearson’s correlation analysis showed that there was no correlation between most of the environmental parameters and the abundance of anammox bacteria. However, the sediment median size positively correlated with the abundance of anammox bacteria (27).

## Discussion

### Anammox bacterial diversity and distribution in sediments from marginal seas

Some previously used anammox bacterial-specific primers were employed in this study according to the defined PCR protocols (1, 36, 37). These primers included An7F-An1388R and Amx368F-Amx820R; however, only limited sites got positive results. Few studies have proposed that the diversity of anammox bacteria may be improved and some novel assemblages related to anammox bacteria unveiled with more specific primers and improved PCR conditions (1, 5). A nested PCR strategy was preferred for 16S rRNA and *hzo* gene sequences to construct gene libraries in order to obtain better results and acquire comprehensive information on the diversity, community structure, and distribution of anammox bacteria in the sediments of such diverse ecosystems. Although a single genome of anammox bacteria may contain many copies of the *hzo* gene (44), which will confound diversity patterns in molecular studies, the *hzo* gene is still a good complement to the 16S rRNA gene because it offers significant information on the potential role of anammox bacteria in marine ecosystems (15). In addition, the other functional marker, the hydrazine synthase gene, is less targeted and has less available sequences affiliated to the *Scalindua* clade, the most common group of anammox bacteria in the marine environment.

Anoxic/oxygen minimum zones are appropriate and suitable locations for the growth of highly diverse anammox bacteria (20). Using both gene markers, the diverse phylotypes of anammox bacteria were detected in the present study, congruent to previous findings obtained in sediments from BS, hypernutrified Jiaozhou Bay, and Pearl Estuary (6, 7, 12). Specifically, the *Scalindua* group was found to be highly dominant in all sites by both primers. Diverse guilds of anammox bacteria (eight novel clades) not described before were detected with 16S rRNA gene sequences. Of these, two novel clades were closely related to known anammox genera according to the results of the similarity analysis and high bootstrap values in the phylogenetic tree. These results showed the high diversity of the anammox lineage in the unusual microenvironment of the north marginal seas of China. The prevalence of the *Scalindua* lineage with both gene biomarkers validated members of *Scalindua* being major anammox bacteria in marine environments and suggests that they play a significant role in nitrogen production. Some of the sequences retrieved from sites closer to land were similar to those recovered from the wastewater treatment plant treating landfill leachate in Pitsea, UK (36), indicating that, due to close proximity, these sampling sites are more prone to terrigenous contamination.

The sites BS1 and BS3 were found to harbor the most diverse anammox community. These sites were located in BS, which receives a huge amount of river discharge and various terrestrial and anthropogenic materials such as sediment, nutrients, and contaminants (6). Hence, the frequent influence of nearby land and human activities may play a prominent role in enhancing the anammox biodiversity of these sites. 16S rRNA gene primers revealed that “*Ca.* Scalindua marina” and “*Ca.* Scalindua pacifica” were the most dominant species (almost 33% and 23%) among the anammox lineages throughout the studied sites, particularly from BS. “*Ca.* Scalindua brodae/sorokinii” each accounted for 15%, whereas the seldom detected “*Ca.* Scalindua wagneri” and “*Ca.* Scalindua Arabica” were estimated to account for 5.5% and 8.5%, respectively, from all gene libraries. More than 50% of the novel clades were recovered from NECS and 30% from SYS. These two neighboring areas shared more or less similar ecological parameters including DO, salinity, Chl *a*, and sediment median size, making them relatively different from the two other marginal seas (27); consequently, we assumed that these distinctive ecological features may promote the prevalence of novel clades in sediment from these seas. Total organic matter and salinity were identified as environmental factors affecting the distribution of anammox bacteria throughout the sites studied. The *Scalindua* genus of anammox bacteria are dominant in many environments with high salinity, partly due to its high tolerance and adaptability to salinity, and this was also the case in the present study in which the high diversity of the *Scalindua* group was detected. However, the detection of the *Jettenia* and *Anammoxoglobus* clades by *hzo* gene primers within the high salinity zones also indicated their potential capabilities to survive under these conditions, but in a limited quantity. Furthermore, total organic matter in sediment across all sites was lower than that reported previously. A low organic carbon content was proven to be one of the significant ecological factors expanding the diversity and community composition of anammox bacteria in marine sediments (9), and previous studies verified that a low amount of organic matter may enhance the prevalence of the anammox bacterial community (9).

Sediment form NECS2 displayed the most diverse anammox bacterial community in terms of *hzo* gene sequences, and the smaller amount of total organic matter and water content from this site (27) may be shaping increased anammox diversity as described previously (9). Similarly, BS1 was identified as the location with the second highest anammox diversity, as shown by the diversity indices and number of OTUs. A relatively high diversity of anammox bacteria was detected in sediment from BS and NECS with the gene markers used in the present study. These gene markers revealed the higher diversity of anammox bacteria in sediment from BS and NECS than in those from the other sites studied. However, similar to the results obtained using 16S rRNA gene sequences, low diversity was estimated from the SYS3 sampling site of SYS (27).

Based on *hzo* gene sequences, temperature appeared to be the only prominent factor shaping anammox bacterial distribution among all the environmental parameters analyzed. Previous studies reported that *Scalindua* was the most dominant and prevalent anammox bacteria in low temperature water columns or surface or subsurface marine sediments (6, 26). The distribution pattern of the anammox community revealed by both gene markers indicated that some of the sites displayed dissimilar biogeographical distributions; however, it was not possible to distinguish most sampling sites into a separate geographical group and they shared similar anammox bacterial assemblages (Fig. S3 and S4).

### Phylogeny by 16S rRNA gene and *hzo* gene sequences

During the study with 16S rRNA genes, only members of *Scalindua* were identified among known genera of anammox bacteria. This may have been due to the lack of non-*Scalindua* anammox bacteria or the detection limit of the 16S rRNA gene primers used and bias of the PCR assay (1, 5, 32). To date, nine distinctive members of the genus *Scalindua* have been documented in various studies (3, 6, 36), and most of the known members were detected in our study. Using the 16S rRNA gene sequences, two novel clades were detected from the sediments of NYS and BS, and may belong to novel members of anammox bacteria (Fig. 2). Distance-based sequence similarities with all defined anammox sequences, Blast results in the NCBI, and the unique position in the phylogenetic tree with high bootstrap values confirmed the novelty of these clusters. Cluster II contained two phylotypes and they shared a sequence similarity of less than 95% with each other. However, we were unable to confirm whether these are novel anammox genera unless further evidence regarding their anammox potential is obtained.

Most of the novel clades chiefly belonged to BS and NECS, validating the ecologically diverse nature of these studied sites. Moreover, some of the closest matches of certain novel sequences were retrieved from diverse environmental samples with different origins; hence, we presumed that the environment studied has enormous anammox bacterial diversity. Novel clades I to VI in the consensus tree of the 16S rRNA gene were considered to be new members of *Planctomycetes* due to their ambiguous phylogenetic positions, pairwise distance similarity, and closest match results obtained through the GenBank database. Although novel clusters IV and VI were assembled with the recently described novel members of *Ca.* Anammoxidans sediminis and *Ca.* Aestuarianus humenicus retrieved from sediment in Pearl Estuary (12), they showed relatively low identity (<75%) with all the known genera of anammox bacteria. Due to these ambiguities, we did not presume the two clusters to be novel anammox groups. Metagenomic approaches with the ability to resolve the phylogeny, ecophysiology, and biogeochemical functions of anammox bacteria from natural environments (50) are, thus, of great value in further studies.

Using *hzo* gene primers, the *Scalindua* lineage was found to be the most prevalent and dominant anammox genus throughout the sites studied. The identification of novel clades with both gene markers in the sediments of the selected sites, particularly NECS, confirmed that they had distinguished anammox community and composition with exceptional diversity and abundance, which may have been underestimated in previous studies. This is mainly attributed to nutrient pressure resulting from excessive anthropogenic activities from the last few decades.

Moreover, different from the 16S rRNA genes, non-*Scalindua* genera were also discovered with *hzo* genes in sediment from adjacent sites, *i.e.*, *Ca.* Jettenia from SYS3, which is relatively close to the coastal area of Jiangsu province, and *Ca.* Anammoxoglobus from NECS1, which is close to the Yangtze estuary. *Ca.* Anammoxoglobus may be shifted through terrestrial contamination by the estuary. In a previous study, *Jettenia* clade was also recovered with *hzo* gene primers from the site near to the coastal area of YS (6). In this context, the *Jettenia* clade may be recovered due to the close proximity of the site (SYS3) with the nearby coastal area of Jiangsu, which may be the possible entry source. Some of the anammox bacteria may tolerate adverse environmental conditions while others cannot; therefore, non-native marine anammox bacteria may survive in marine environments in a limited quantity with undetectable anammox activity (1). It was also deduced by comparisons with previous studies (25, 33) that the 16S rRNA and *hzo* gene primers have dissimilar lineage coverage for anammox bacteria.

### Anammox bacterial abundance and community structure

16S rRNA gene primers revealed that the anammox population in the study area was abundant in most of the sites studied. Abundance was higher in sediment from SYS2 and SYS3, estimated to be 9.21×10^5^ and 9.20×10^5^ copies g^−1^ wet sediment, respectively. However, the lowest abundance of anammox bacteria was determined to be 3.95×10^5^ copies g^−1^ wet sediment in SYS1. Furthermore, the relatively low diversity but high abundance of anammox bacteria in sites from SYS indicated that the role of anammox bacteria in the nitrogen cycle is not necessarily linked to anammox diversity.

The abundance of anammox bacteria was lower than previously documented in sediments from BS, Dongjiang River, and the Yangtze Estuary (6, 17, 45), whereas other studies indicated the higher magnitude of anammox bacteria in surface sediment from the Equatorial Pacific and water samples from Shengli Oilfield than that in the present study (18, 24). However, the results obtained were consistent with sediment samples from hypernutrified Jiaozhou Bay, which ranged between 6.22×10^5^ and 4.86×10^6^ using anammox 16S rRNA gene primers (7). The median size of the sediment sample showed a positive correlation with the abundance of anammox bacteria (Table S2).

The results retrieved through environmental clustering by the Jackknife method and PCoA analysis with both gene markers indicated heterogeneity among the anammox assemblages of a few sites; however, most of the other studied sites showed undistinguished anammox bacterial assemblages (Fig. S3 and S4).

## Conclusions

To our best knowledge, this is the first time different areas of the north marginal seas in China were covered to analyze the diversity, pattern of distribution, and abundance of anammox bacteria in a single study. *Scalindua* was the most dominant clade across all the sites examined. In addition, two potential novel anammox groups and six *Planctomycetes* members were detected, indicating potentially high anammox bacterial diversity in the north marginal seas of China. Salinity, temperature, and total organic matter were confirmed to be the key regulators shaping the anammox community structure and distribution in the coastal marine sediment; however, the role of other as yet unidentified ecological parameters cannot be ruled out. The high abundance of the anammox bacterial community in sediment from SYS may indicate their significant roles in the nitrogen cycle among the investigation sites. However, further efforts are needed in order to identify hidden anammox communities, particularly by designing targeted primers with high coverage for functional and 16S rRNA genes along with specific PCR profiles. Due to the limited ecological datasets used in the present study, various additional physicochemical profiles need to be determined in order to obtain a better understanding of their actual roles in shaping the anammox bacterial community structure, distribution, and abundance in a more comprehensive manner from such a diverse ecosystem, thereby exposing these ambiguities.

## Supplementary Material



## Figures and Tables

**Table 1:** Diversity and calculated richness of anammox bacteria based on the 16S rRNA gene and *hzo* gene sequences recovered from selected sites in north marginal seas in China.

Station ID	No. of sequences		No. of OTUs		Shannon		Chao1	
			
	16S	*hzo*	16S	*hzo*	16S	*hzo*	16S	*hzo*
NYS1	41	54	7	5	1.211	0.795	8	6
NYS2	46	46	3	10	0.739	1.455	3	31
NYS3	38	35	6	5	1.149	0.871	6.5	8
BS1	39	38	12	9	2.214	1.956	12.6	11
BS2	45	63	4	11	0.628	0.804	4	18.5
BS3	36	42	10	4	1.889	1.097	10.2	4
NECS1	34	36	4	9	0.632	1.726	5	12
NECS2	34	38	6	12	1.518	1.986	6	22.5
NECS3	34	40	6	2	1.517	0.233	6	2
SYS1	41	38	8	6	1.872	0.763	9	7
SYS2	44	54	8	7	1.707	1.164	8.5	8
SYS3	44	45	2	3	0.507	0.222	1.5	4
