# Supplementary material for: Diversity, Community Composition and Abundance of Anammox Bacteria in Sediments of the North Marginal Seas of China
Source: Microbes Environ. 2016 May 14;31(2):111–20. doi: 10.1264/jsme2.ME15140 (PMC4912145; doi:10.1264/jsme2.ME15140)
Supplement: Supplementary file 1 [file 31_111_s1.pdf]

Supplementary materials for

Diversity, community composition and abundance of anammox bacteria in sediments of the north marginal seas of China

Ahmed Shehzad, Jiwen Liu, Min Yu, Shakeela Qismat, Jingli Liu, Xiao-Hua Zhang

Table S1 Determination of sensitivity and efficiency of qPCR standard curve by using linearized plasmid DNA.

Target gene	primer	Efficiency			Sensitivity (Anammox 16S rRNA gene) copies g^{-1} sediment	
		R^2 linearity	slope	range		
Anammox bacterial 16S rRNA gene	AMX-808F AMX-1040R	0.996	-3.0829	10^1-10^6	2.3X10^5	3.55X10^5

Table S2 Correlation analyses of diversity and abundance of anammox bacteria community with available environmental factors

factors	Gene biodiversity		Gene abundance
	hzo gene biodiversity	16S rRNA gene biodiversity	
TOM	0.6800	**0.0080**	0.4351
Water contents	0.0760	0.7840	0.2789
Sediment median size	0.0640	0.2420	**0.07967**
pH	0.6680	0.4000	0.2724
Salinity	0.3940	**0.0440**	0.1275
Dissolved oxygen	0.3680	**0.0520**	0.9967
Temperature	**0.0240**	0.5780	0.2521
Chla	0.0740	0.1040	0.2571

The environmental factors have positive correlation with anammox community indicated with bold font.

Table S3 Results representing abundance of anammox bacteria from all the selected sites of the north marginal seas

**Samples ID**	**(Anammox 16S rRNA gene) copies g^{-1} sediment**
NECS1	$4.94x10^{5}$
NECS2	$6.86x10^{5}$
NECS3	$4.01x10^{5}$
BS1	$8.18x10^{5}$
BS2	$5.51x10^{5}$
BS3	$4.95x10^{5}$
NYS1	$8.30x10^{5}$
NYS2	$7.96x10^{5}$
NYS3	$4.78x10^{5}$
SYS1	$3.95x10^{5}$
SYS2	$9.21x10^{5}$
SYS3	$9.20x10^{5}$

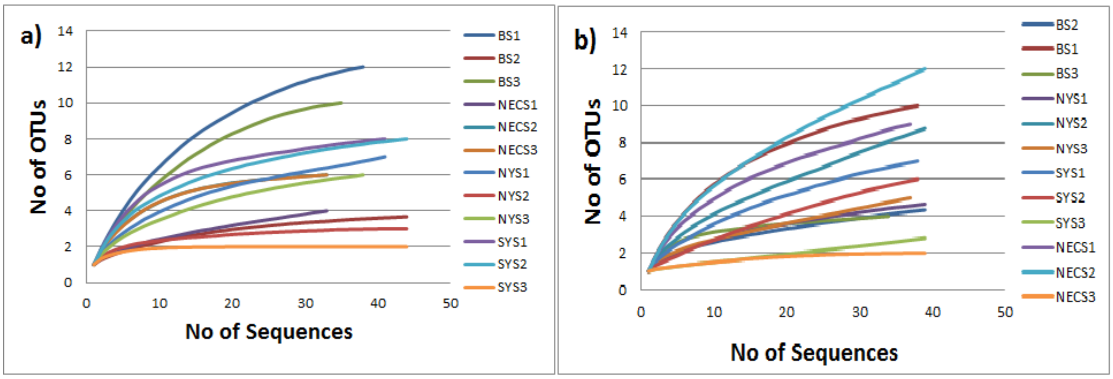

Fig. S1 Rarefaction analysis of anammox bacterial community with a) 16S rRNA & b) Hzo protein sequences from the sediments of north Marginal Seas of China by using DOTUR program with 3 % cutoff value.

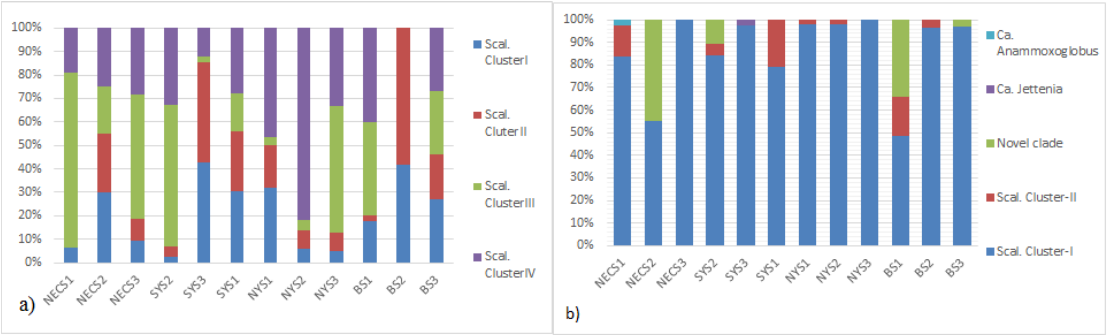

Fig. S2 Community structure of anammox bacteria from the sediments of north marginal China seas using (a) 16S rRNA gene libraries and (b) *hzo* gene libraries. Abbreviations: ana, anammox; Planc, Planctomycetes; Scal, Scalindua.

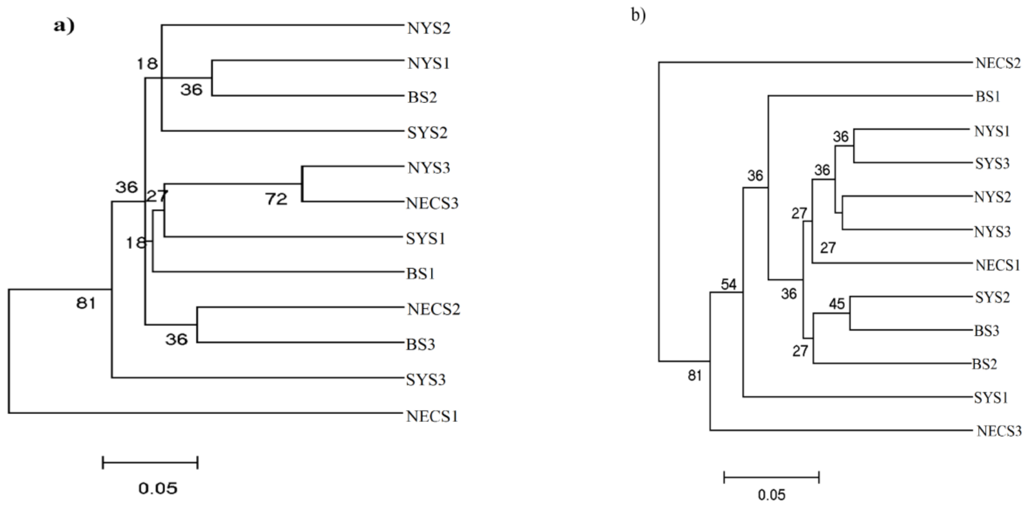

Fig. S3 Dendrograms of the hierarchical clustering analysis of sediment of north marginal seas of China by using 16S rRNA (a) and *hzo* gene sequences (b). The clustering dendrograms were constructed by using UniFrac weighted Jackknife Environment Clusters statistical method.

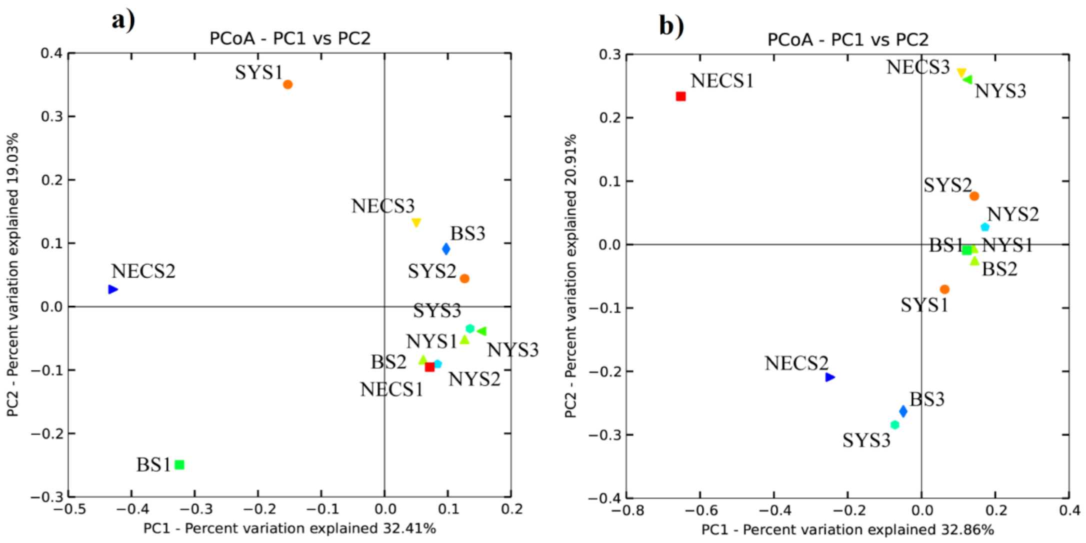

Fig. S4 Principal coordinate plot by UniFrac analysis of (a) 16S rRNA gene and (b) Hzo protein sequences from 12 marine sediments samples collected from four north marginal seas of China.
